# Structural Alterations and Cognitive Impairment in Late-Onset Depression: A Reverse Correlation Analysis

**DOI:** 10.31083/AP44585

**Published:** 2026-02-03

**Authors:** Yujie Wu, Naikeng Mai

**Affiliations:** ^1^School of Education Science, Guangdong Polytechnic Normal University, 510665 Guangzhou, Guangdong, China; ^2^Department of Psychosomatic Medicine, Guangdong Provincial People's Hospital, 510080 Guangzhou, Guangdong, China; ^3^Department of Neurology, The Affiliated Brain Hospital of Guangzhou Medical University (Guangzhou Huiai Hospital), 510370 Guangzhou, Guangdong, China

**Keywords:** depression, cognitive impairment, machine learning, neural networks, magnetic resonance imaging

## Abstract

**Background:**

Late-onset depression (LOD), particularly when accompanied by cognitive impairment, represents a significant risk factor for dementia. Prevailing perspectives emphasize that cognitive impairment arises from interactions among multiple brain regions. However, current approaches to identifying brain network patterns associated with cognitive impairment largely rely on group-level analyses with multiple-comparison corrections, which may obscure complex and interconnected relationships between brain regions. Our previous research demonstrated that alterations in brain network properties in patients with LOD are closely associated with cognitive function. We therefore hypothesised that aberrant interactions among multiple brain regions in LOD lead to changes in network properties and subsequent cognitive dysfunction.

**Methods:**

This study aimed to investigate the interregional brain interactions underlying cognitive impairment in LOD by leveraging the robust interpretability of neural network models. Specifically, we sought to: (1) develop a neural network model of LOD-related cognitive impairment based on brain network properties; and (2) apply a reverse correlation approach to identify connectivity features associated with cognitive impairment in LOD.

**Results:**

No statistically significant differences were observed in tthe structural network properties when comparing the LOD and control participant groups across various thresholds. Using a neural network–based reverse correlation method, the most prominent differences were identified in the inferior, middle, and anterior regions of the left temporal pole when comparing patients with LOD with and without mild cognitive impairment (MCI).

**Conclusion:**

Alterations in the internal structure of the temporal lobe may represent potential anatomical biomarkers for the early prediction of Alzheimer’s disease, providing novel insights into the pathophysiological mechanisms underlying LOD-related MCI. The research framework proposed in this study effectively addresses the challenge of detecting subtle intergroup anatomical differences in studies with limited sample sizes. Moreover, the reverse correlation approach is not restricted to multilayer neural networks; as machine learning models become increasingly powerful and accessible, this method offers a practical and interpretable alternative for exploratory neuroimaging research.

## Main Points

1. Patients with late-onset depression and mild cognitive impairment exhibit specific structural alterations in brain regions.

2. The neural network and reverse correlation-based approach effectively identifies subtle intergroup differences.

3. Temporal lobe structural alterations may reflect an early stage in the transition from late-onset depression to cognitive impairment.

## 1. Introduction

With an ageing population, the healthcare burden related to late-life conditions continues to rise. Late-life depression (LLD), defined as depression occurring in adults aged 60 and above, is one of the most common mental disorders among older adults. The risk of depression increases with age in this group. LLD is a known risk factor for Alzheimer’s disease and vascular dementia [1], and when coexisting with mild cognitive impairment (MCI), it significantly predicts dementia [2, 3, 4]. LLD can be divided into early-onset depression, which recurs or persists into old age, and late-onset depression (LOD), characterised by a first depressive episode occurring at 60 or older. early-onset depression may be associated with genetic predisposition, a family history of mental illness, and stress-related factors [5, 6]. Our recent study identified distinct differences between early-onset depression and LOD in dynamic network modularity and rich-club node distribution, assessed via resting-state functional magnetic resonance imaging [7]. These differences correlated with cognitive function in only one subtype, suggesting distinct mechanisms of cognitive impairment in early-onset depression and LOD. Given that LOD poses a higher risk for cognitive decline than early-onset depression, this study focused on analysing the structural correlates of cognitive impairment specifically in LOD.

Graph theory enables the quantitative analysis of brain network topology. The key metrics in this respect include: global efficiency; nodal strength; clustering coefficient; shortest path length; and local efficiency. These metrics capture how efficiently information is integrated both globally and locally, and relate closely to cognitive abilities and clinical symptoms. Rich-club properties and modularity further describe the brain’s balance between integration and segregation.

Our previous work demonstrated synergistic effects of LLD and MCI on brain ageing. Structural covariance networks revealed that grey matter alterations in LLD-MCI resemble those observed in Alzheimer’s disease [8]. Memory impairment in LLD correlates with white matter rich-club reorganisation, where the connectivity strength of rich-club and local networks, shortest path length, global efficiency, and local efficiency, is associated with cognitive function [9], indicating both grey and white matter involvement in LLD-related cognitive decline. Although functional magnetic resonance imaging (fMRI) studyin LLD-MCI have reported similar network alterations, no cognitive correlations were identified [10]. Our recent dynamic network analysis showed that modularity correlates with memory in early-onset depression but not in LOD, with similar patterns observed in rich-club analysis (see Research Foundation). This suggests distinct structural substrates for cognitive impairment in early-onset depression versus LOD. However, the specific brain regions driving changes in rich-club organization and modularity remain unidentified. Pinpointing the key nodes influencing rich-club organization is crucial for understanding the neuropathology of LLD and for developing targeted neuromodulation therapies, such as Transcranial Magnetic Stimulation/transcranial Direct Current Stimulation.

Advances in methodology have broadened the applications of machine learning [11]. Clinically, machine learning supports diagnosis, prognosis prediction, and the assessment of treatment response [12, 13, 14]. It also enables the quantification of disease-induced neural damage [15]. Our preliminary studies applied machine learning to evaluate brain ageing, confirming synergistic LLD-MCI effects on cognitive decline [8]. Although MRI-derived network properties partially reflect cognitive function in LLD [9], conventional comparisons fail to pinpoint the structural signatures of impairment. In this study, we employed neural networks to investigate the structural and functional features of cognitive impairment in LOD.

Owing to the strong interpretability of back propagation neural networks, a classification model can be established. However, unlike regression models, the presence of hidden layers in back propagation networks makes it challenging to interpret connection weights and classifier thresholds in real-world terms. Nevertheless, their excellent generalisation performance makes back propagation networks a powerful tool for gaining deeper insights into underlying principles of complex systems. To explore their potential as feature-extraction tools in brain networks, we applied the reverse correlation method, using classification results to infer internal representations. Reverse correlation is a data-driven technique for visualising mental representations. In this approach, participants are shown a series of stimuli created by adding random noise patterns to a base image, typically a face with a neutral expression. These variations were generated by superimposing random noise patterns onto the base image [16].

In this study, the neural network model served as the “participant” classifying healthy human brain networks that had been perturbed by the addition of random noise. By integrating these noise patterns, we extracted brain network features associated with cognitive impairment.

Given that interregional brain connectivity underpins higher-order neural functions, this study leveraged the predictive power of neural networks to identify dysregulated connectivity in LOD-related cognitive impairment. Using reverse correlation, we extracted discriminative features from the model to uncover structural network signatures of LOD pathology.

## 2. Methods

This cross-sectional study was conducted at the Brain Hospital affiliated with Guangzhou Medical University, China. This study was approved by the ethics committee of the Guangdong Provincial People’s Hospital and written informed consent was obtained from each participant. The inclusion criteria for patients with LOD were as follows: (1) age ≥60 years; (2) diagnosis of major depressive disorder according to the Diagnostic and Statistical Manual of Mental Disorders, Fifth Edition (DSM-5); (3) confirmation of major depressive disorder by psychiatrists at the Brain Hospital affiliated with Guangzhou Medical University; (4) the ability to complete neuropsychological assessments; (5) first depressive episode occurring at or after the age of 60. The exclusion criteria included: (1) the inability to complete neuropsychological testing; (2) a history of severe psychiatric disorders (e.g., bipolar disorder and schizophrenia) excluding depression; (3) a family history of severe psychiatric disorders (e.g., bipolar disorder and schizophrenia) excluding depression; (4) primary neurological disorders (e.g., stroke and brain tumours); (5) medical conditions potentially affecting mood (e.g., hypothyroidism, syphilis, and anaemia); (6) contraindications for MRI (e.g., non-cooperation or intracorporeal metallic implants); (7) Hamilton rating scale for depression (HAMD) >7; (8) Clinical Dementia Rating (CDR) score >0.5; (9) Hachinski Ischaemic Scale score >4.

Patients with LOD were further stratified into the following groupings:

MCI group: diagnosed using Petersen’s 1999 criteria [17]: (1) self- or informant-reported memory complaints; (2) objective cognitive impairment ≥1.5 standard deviation units below age- and education-matched norms; (3) CDR = 0.5 and Global Deterioration Scale stages 2 and 3; (4) preserved activities of daily living; and (5) absence of dementia.

Cognitively normal group (NON-MCI): patients with LOD not meeting the MCI criteria.

Healthy controls (HCs) met the following criteria: (1) age >60 years with no history of depression; (2) exclusion criteria similar to the LOD group; and (3) CDR ≤0.5 (to exclude dementia-depression comorbidity).

The final study cohort was comprised of the following participants: (1) 42 patients with LOD and 90 HCs from prior studies who met all criteria; (2) one patient with LOD who was excluded owing to incomplete diffusion tensor imaging (DTI) scanning.

All participants underwent clinical assessments, neuropsychological testing, and neuroimaging within 1 week of enrolment.

### 2.1 Clinical Assessments

Diagnostic reviews were conducted by trained clinicians holding intermediate-to-senior professional titles, all certified in the Structured Clinical Interview for DSM-5 Axis I Disorders. Detailed demographic and clinical data were collected from all participants, and comprehensive physical examinations were performed to assess exclusion criteria. Screening for enrollment utilized the CDR, Mini-Mental State Examination (MMSE), and HAMD. The medication history of LOD are presented in **Supplementary Table 1**.

### 2.2 Neuroimaging Data Acquisition

Cranial MRI scans were performed using a 3.0-Tesla MRI system (Siemens Magnetom PRISMA, Siemens Healthcare, Erlangen, Germany) at the Guangzhou Medical University Affiliated Brain Hospital, China. Prior to the formal scanning protocol, T2-weighted imaging was conducted to exclude cerebral infarction, haemorrhage, tumours, malformations, and extensive white matter lesions.

Prior to resting-state functional magnetic resonance imaging scanning, participants were instructed to remain relaxed with their eyes closed while refraining from falling asleep. Resting-state functional magnetic resonance imaging images were acquired using a gradient-echo echo-planar imaging sequence with the following parameters: TR = 2000 ms, TE = 30 ms, FA = 90°, matrix = 64 × 64, slice thickness = 4 mm, slice gap = 0.6 mm, 33 interleaved axial slices, field of view (FOV) = 220 × 220 mm^2^, and 240 time points.

DTI parameters were as follows: 32 directions, *b*-value = 1000 s/mm^2^, TR = 10,015 ms, TE = 92 ms, flip angle = 90°, imaging matrix = 128 × 128, FOV = 256 × 256 mm^2^, 75 contiguous slices, and voxel dimensions = 2 × 2 × 2 mm^3^.

High-resolution T1-weighted images were acquired using a three-dimensional (3D) spoiled gradient-echo sequence with the following parameters: TR = 8.2 ms, TE = 3.8 ms, slice thickness = 1 mm, FOV = 256 × 256 mm^2^, and matrix = 256 × 256 × 188.

### 2.3 Brain Network Construction and Feature Computation

#### Data Preprocessing

DTI preprocessing was performed using the FMRIB Software Library (FSL) Diffusion Toolbox and included the following steps: Eddy current correction: compensated for magnetic field distortions and head motion. (1) b_0_ extraction and skull-stripping: intensity threshold = 0.2. (2) Bayesian Estimation of Diffusion Parameters Obtained using Sampling Techniques (BedpostX): generated whole-brain voxel-wise fibre orientation distributions. (3) T1 skull-stripping: intensity threshold = 0.3.

### 2.4 Network Construction

Node Definition: Using the Desikan–Killiany atlas, the following steps were performed: (1) T1 structural images were non-linearly registered to the MNI152 standard brain template (based on 3D MRI scans of 152 healthy individuals), producing an inverse warp file. (2) The Desikan–Killiany atlas was then transformed from T1 space to b0 space using the corresponding transformation matrix. (3) This resulted in 82 regional masks registered to each subject’s DTI space. Each regional mask served as a seed, while the remaining 81 masks were treated as terminal masks. Edge definition: probabilistic fibre tracking (FSL 5.09) was employed to define network edges. For each voxel within a seed mask, 5000 streamlines were generated. Streamline propagation followed the orientation distributions estimated by BedpostX, advancing in 0.5-mm steps toward the 81 target masks. Tracking stopped upon reaching a target mask to prevent infinite loops caused by circular fibres.

The connection probability between the seed mask and each target mask was calculated and used as the connection weight between nodes. After tracking was completed for all 82 seed masks, an 82 × 82 connectivity matrix representing individual white matter structural connectivity was generated. As probabilistic tracking does not provide directionality, the connection weights between nodes *i* and *j* (weight_ij_ and weight_ji_) were used to produce an undirected connection weight.

### 2.5 Network Calculation

Network properties were computed across multiple connection density thresholds using custom scripts. The area under the curve (AUC) for each property across thresholds represented the overall network characteristic of the participant. The following parameters were calculated in this study: clustering coefficient (*aC*p), global efficiency (*aE*g), local efficiency (*aE*loc), characteristic path length (*aL*p), modularity (*a*Q), rich-club connection strength (*Rich_s*), local connection strength (*Local_s*), and feeder connection strength (*Feeder_s*). Equations and detailed interpretations have been provided in our previous study [9].

### 2.6 Construction of a Neural Network Model for Cognitive Impairment Based on Brain Network Features

Features including *a*Q, *Local_s*, *Rich_s*, *aE*g, *aE*loc, and *aL*p were computed for both early-onset depression and LOD groups using the Brain Connectivity Toolbox (https://sites.google.com/site/bctnet/home) and custom Matrix Laboratory (MATLAB) (2024b, https://ww2.mathworks.cn/products/matlab.html) scripts. These features were then used to train a cognitive prediction model. Labels (cognitively normal vs. impaired) were assigned according to Petersen’s 1999 MCI diagnostic criteria.

The neural network consisted of three hidden layers with six units each. Data were randomly split into training, testing, and validation sets in an 8:1:1 ratio. Implemented using the Neural Network Toolbox (3.0, The MathWorks, Inc., Natick, MA, USA) of MATLAB, the model employed: (1) training algorithm: scaled conjugate gradient; (2) performance metric: cross-entropy; and (3) given the limited feature set, no regularisation was applied to hidden layers.

The 3D distribution of real versus predicted data and the receiver operating characteristic curve for the LOD cognitive impairment classification model are presented in **Supplementary Figs. 1,2**.

#### 2.6.1 Standard Network Construction

The structural connectivity matrices from 90 cognitively normal older participants were used to construct an edge-weight distribution curve for the HC group. After removing the top and bottom 2.5% of values, the mean of the remaining edge weights was calculated to establish a reference matrix for healthy ageing brain networks.

#### 2.6.2 Randomisation Procedure

**Fig. 1.**
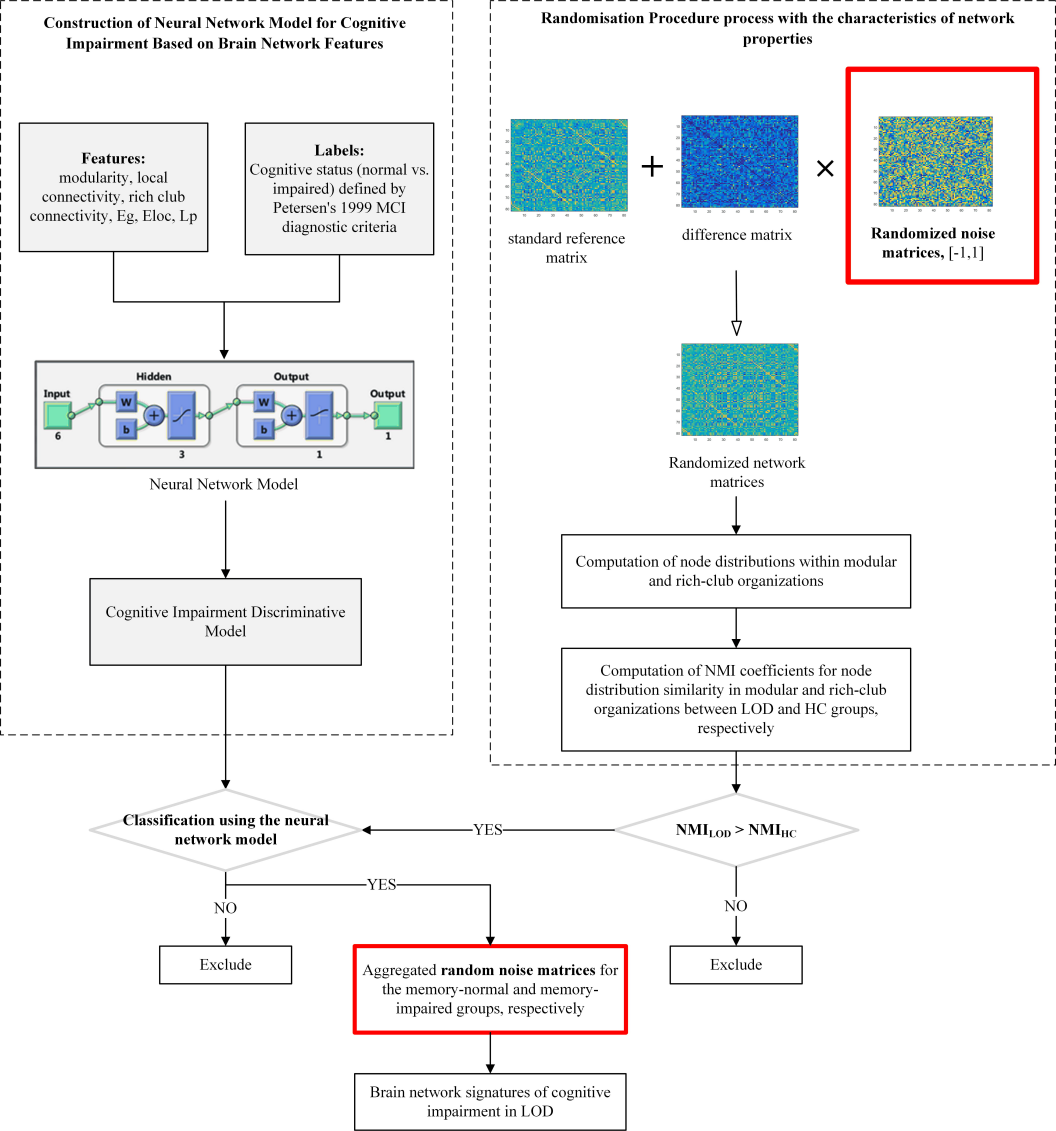
**Flowchart of the study on network characteristics of LOD with cognitive impairment using randomisation procedure and reverse correlation method**. The red box represents the data source of the final result, which is a synthesis of random matrix data filtered by the discriminant model. LOD, Late-onset depression; MCI, mild cognitive impairment; NMI, normalised mutual information; HC, Healthy control.

A difference matrix was generated by comparing the data of the LOD with that of the HC group. This difference matrix was randomised using the procedure depicted in Fig. 1 to produce randomised network matrices. Modular and rich-club partitions were then performed, and the normalised mutual information (NMI) for these partitions was calculated for both LOD and HC configurations derived from the randomised matrices. Randomised matrices satisfying the criterion NMI_LOD_ < NMI_HC_ were selected for further analysis. The equation used to compute NMI is given below.

$$N\,M\,I\,\left(A\,1,A\,2\right)=\frac{-2\,\sum_{i=1}^{C_{A\,1}}\sum_{j=1}^{C_{A\,2}}N_{i\,j}\,\log\left(\frac{N_{i\,j}\,N}{N_{i}\,N_{j}}\right)}{\sum_{i=1}^{C_{A\,1}}N_{i}\,\log\left(\frac{N_{i}}{N}\right)+\sum_{j=1}^{C_{A\,2}}N_{j}\,\log\left(\frac{N_{j}}{N}\right)}.$$

In the normalized mutual information (NMI) calculation, A1 and A2 represent the modular and rich-club partitions from the randomised networks and the LOD (or HC) network, respectively. CA1 and CA2 indicate the number of partitions in A1 and A2. N_ij_ is the number of nodes shared between a partition in A1 and one in A2. The total number of nodes, N, is 82 in this study. N_i_ and N_j_ refer to the number of nodes within a partition of A1 and A2, respectively. A higher NMI value reflects greater similarity between the modular and rich-club partitions of the randomised and LOD (or HC) networks.

#### 2.6.3 Reverse Correlation for Cognitive Impairment Network Signatures

A total of 10,000 randomised networks were generated. (1) The NMI coefficient was calculated based on network properties and compared with the NMI coefficients of LOD and HC in the actual samples. Randomised networks resembling the network properties of LOD were retained. (2) A pre-trained neural network model was then used to classify these retained randomised networks, extracting those identified as MCI or NON-MCI. (3) Randomised difference matrices were derived from the networks classified in step 2. Aggregating these matrices revealed network connections associated with MCI or NON-MCI risk. To identify meaningful differences within the limited sample size, the top 5% most frequently occurring connections were selected as potential LOD network features related to MCI or NON-MCI.

### 2.7 Statistical Analysis

Differences in age, HAMD, MMSE, and CDR between patients with LOD and HCs were analysed using the independent-samples *t*-tests. Differences in sex distribution between groups were assessed using the Chi-square test. Group differences in structural network properties were evaluated using multivariate analysis of covariance, controlling for age as a covariate. For demographic data that did not pass the Levene’s test, the Wilcoxon rank-sum test was employed. For group differences in structural network characteristics that violated normality assumptions, the ranks of covariates and dependent variables were first calculated. Linear regression was then used to adjust the ranks of the dependent variable based on the ranks of the independent variable, followed by the Kruskal–Wallis test to compare the adjusted residuals. The significance level was set at α = 0.05, with *p* < 0.05 considered statistically significant.

## 3. Results

### 3.1 Demographic Characteristics

No statistically significant difference in age and sex distribution was observed between the LOD and HC groups (*p* > 0.05). However, significant differences were found in the HAMD, MMSE, and CDR scores between the two groups (*p* < 0.05). No statistically significant difference in age, sex distribution, HAMD, MMSE, and CDR scores was observed between MCI and NON-MCI subgroups within the LOD (*p* > 0.05). The age of onset of LOD was 65.98 ± 5.99. Detailed data are presented in Tables 1,2.

**Table 1.** **Demographic characteristics of LOD vs. HC groups**.

	Age (years)	Sex (male/female)	HAMD	MMSE	CDR
LOD (*n* = 41)	68.09 ± 6.93	11/30	1.46 ± 2.15	27.26 ± 1.69	0.18 ± 0.24
HCs (*n* = 90)	69.93 ± 6.40	32/58	8.76 ± 7.73	24.34 ± 3.68	0.40 ± 0.20
*t/z/χ^2^*	*t* = –1.441	χ^2^ = 0.972	*z* = –6.416	z = –4.646	z = –4.634
*df*	129	1	129	129	129
*p*	0.152	0.324	0.001	0.001	0.001

HAMD, Hamilton rating scale for depression; MMSE, Mini-Mental State Examination; CDR, Clinical Dementia Rating.

**Table 2.** **Demographic characteristics of MCI vs. NON-MCI subgroups within the LOD**.

	Age (years)	Sex (male/female)	HAMD	MMSE	CDR
MCI (*n* = 21)	70.62 ± 6.92	5/16	10.29 ± 7.85	23.33 ± 3.61	0.45 ± 0.15
NON-MCI (*n* = 20)	69.20 ± 5.90	6/14	7.15 ± 7.46	25.40 ± 3.53	0.35 ± 0.24
*t/z/χ^2^*	*t* = 0.705	χ^2^ = 0.200	*t* = 1.309	t = –1.852	z = –1.633
*df*	39	1	39	39	39
*p*	0.485	0.655	0.198	0.072	0.102

NON-MCI, cognitively normal group.

### 3.2 Structural Network Metrics in LOD

No statistically significant differences were found in the structural network properties between the LOD and HC groups. Detailed data are presented in Table 3.

**Table 3.** **Structural network metrics in LOD**.

Network properties	*aC*p		*aE*g		*aE*loc		*aL*p		*a*Q		*Rich_s*		*Local_s*		*Feeder_s*	
Group	LOD	HCs	LOD	HCs	LOD	HCs	LOD	HCs	LOD	HCs	LOD	HCs	LOD	HCs	LOD	HCs
*n*	41	90	41	90	41	90	41	90	41	90	41	90	41	90	41	90
M ± SD	0.110 ± 0.009	0.111 ± 0.007	0.104 ± 0.004	0.103 ± 0.009	0.144 ± 0.007	0.144 ± 0.009	1.057 ± 0.040	1.071 ± 0.108	0.146 ± 0.010	0.149 ± 0.014	0.626 ± 0.016	0.628 ± 0.021	0.473 ± 0.033	0.479 ± 0.035	0.569 ± 0.022	0.572 ± 0.025
*F*	0.252		0.322		0.114		0.815		1.222		0.208		0.738		0.304	
*p*	0.617		0.571		0.736		0.368		0.271		0.649		0.392		0.582	
FDR	0.736		0.736		0.736		0.736		0.736		0.736		0.736		0.736	

M ± SD, mean ± standard deviation; *aC*p, clustering coefficient; *aE*g, global efficiency; *aE*loc, local efficiency; *aL*p, characteristic path length; *a*Q, modularity; *Rich_s*, rich-club connection strength; *Local_s*, local connection strength; *Feeder_s*, feeder connection strength.

When comparing network features between MCI and NON-MCI subgroups within the LOD group, no statistically significant differences were detected in the AUC values of structural network properties across thresholds. Detailed data are presented in Table 4.

**Table 4.** **Structural network metrics between MCI and NON-MCI subgroups within the LOD group**.

Network properties	*aC*p		*aE*g		*aE*loc		*aL*p		*a*Q		*Rich_s*		*Local_s*		*Feeder_s*	
Group	MCI	NON-MCI	MCI	NON-MCI	MCI	NON-MCI	MCI	NON-MCI	MCI	NON-MCI	MCI	NON-MCI	MCI	NON-MCI	MCI	NON-MCI
*n*	21	20	21	20	21	20	21	20	21	20	21	20	21	20	21	20
M ± SD	0.111 ± 0.008	0.109 ± 0.009	0.104 ± 0.003	0.103 ± 0.005	0.143 ± 0.005	0.144 ± 0.008	1.054 ± 0.032	1.061 ± 0.047	0.145 ± 0.009	0.147 ± 0.012	0.626 ± 0.017	0.627 ± 0.016	0.474 ± 0.031	0.472 ± 0.035	0.569 ± 0.020	0.568 ± 0.023
*F*	0.411		0.202		0.110		0.204		0.201		0.018		0.034		0.044	
*p*	0.525		0.656		0.742		0.654		0.656		0.895		0.855		0.835	
FDR	0.895		0.895		0.895		0.895		0.895		0.895		0.895		0.895	

### 3.3 Node-Level Features in LOD Extracted via Reverse Correlation

**Fig. 2.**
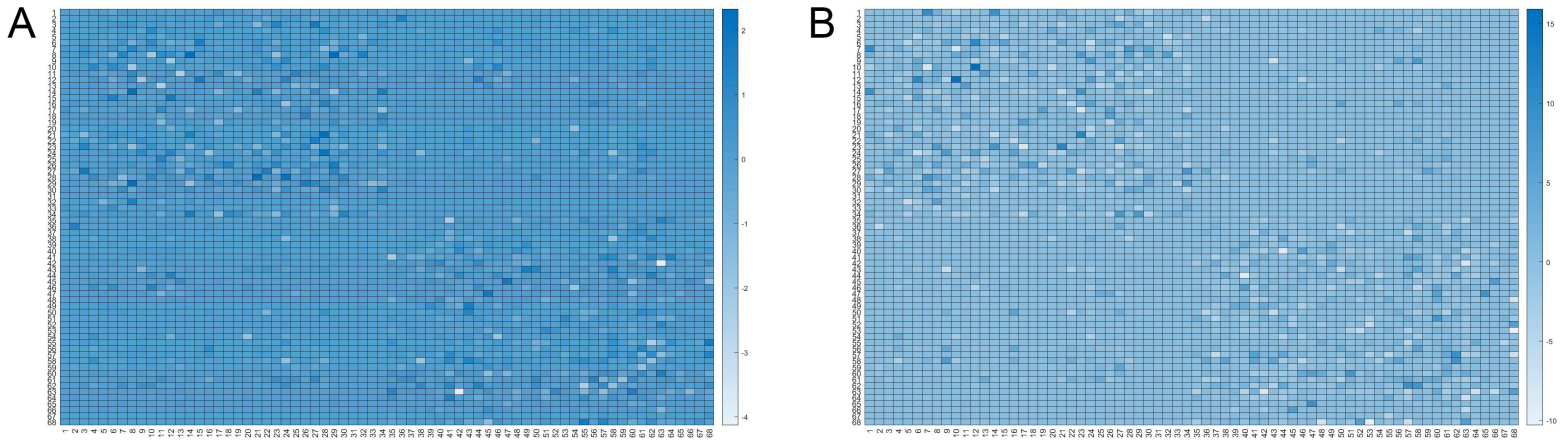
**Matrix visualisations depict the mean of difference node matrices derived from randomisation cycles (n = 10,000)**. For LOD-MCI, the mean of 2645 difference matrices classified as having cognitive impairment was used to generate visualisation (A). Similarly, for LOD non-MCI, the mean of 118 cognitively impaired-classified difference matrices was used to produce visualization (B). The color represents the frequency, ranging from white to dark blue, where a darker shade indicates a higher occurrence rate of the connection in the selected random matrices. Detailed node region information is provided in **Supplementary Material 1**.

For the LOD-MCI subgroup, 10,000 randomisation cycles produced a difference node matrix, resulting in 2645 significant difference matrices. The mean of these was used to produce the matrix visualisation shown in Fig. 2A. Similarly, for the LOD-NON-MCI subgroup, 10,000 randomisation cycles resulted in 118 significant difference matrices, and the mean of these was used to generate the matrix in Fig. 2B.

A comparison of significant random matrix counts revealed that the structural networks in the LOD-MCI group exhibited the most pronounced deviations from the HC group. The most substantial differences were localised in the left inferior, middle, and anterior temporal pole regions. Detailed illustrations are provided in **Supplementary Material 2**.

## 4. Discussion

This study established a standard brain network matrix for healthy ageing using DTI structural images from 90 HCs. By integrating randomised matrices with the difference network between LOD and HC groups and combining them with the standard matrix, we generated random networks constrained within known LOD connectivity parameters. By employing feature comparison with established LOD networks and neural network filtering, we identified LOD-characterised random networks. An aggregation of these matrices revealed distinct network features for LOD-MCI and LOD-NON-MCI. The core innovation of the present study lies in applying reverse correlation to identify those network features that determine training efficiency in well-trained neural networks. This study proposed the application of the reverse correlation method as a tool for interpreting machine learning and identifying potential discrepancy trends in preliminary experiments with limited sample sizes. We aimed to detect divergence trends in sample collection at an early stage in the research, analyse experimental samples based on research objectives, and promptly adjust relevant conditions for sample collection.

No structural network differences were observed between the LOD-MCI and LOD-NON-MCI subgroups. However, our previous brain-age prediction studies had identified significant disparities, suggesting that conventional methods fail to capture abnormalities in clinically distinct cohorts. In contrast, the reverse correlation-based neural network approach successfully detected differential inter-nodal connections. Although statistical validation is still pending, these preliminary results highlight the need for larger-scale investigations.

Matrix significance comparisons revealed the greatest structural network deviations between LOD-MCI (2645/10,000 matrices) and HC groups, and these findings are consistent with the results of prior research. The left inferior, middle, and anterior temporal pole showed the most pronounced differences, suggesting that disrupted intra-temporal white matter connectivity may serve as a potential anatomical biomarker to distinguish HC from LOD-MCI. Although studies on LLD-related cognitive impairment have implicated the cingulate cortex, hippocampus, and prefrontal cortex, these regions lack disease specificity, as they also appear in conditions such as frontotemporal dementia [18] and Lewy body dementia [19]. The encodement of memory depends on coordinated hippocampal engagement [20], frontal lobe integration of entorhinal-hippocampal inputs [21], and medial temporal-hippocampal spatial processing [22]. Given that LOD-MCI is a known risk factor for Alzheimer’s disease, and considering the limited sample size of this study, our findings suggest that structural changes within the dominant temporal lobe may reflect an early stage in the transition from LOD to Alzheimer’s disease. These preliminary results may also provide valuable clues for advancing our understanding of the pathogenesis of LOD-MCI.

The structure–function relationships of the human brain manifest as complex networks that enable efficient segregation and integration of information. Neuroimaging-derived connectivity matrices capture these architectures and can be quantified using graph theory, where nodes represent anatomical or functional regions of interest and edges denote structural or functional connections [23, 24]. Network properties have been shown to correlate with cognition and symptomatology: Li *et al*. [25] linked LLD white matter connectivity to processing speed, while another study reported associations between functional network metrics and depression severity [10]. Our replication of *C*p/*E*g differences between LLD and HC groups supports established findings. The absence of significant LOD subgroup differences may be attributable to limited statistical power, whereas machine learning with reverse correlation successfully detected subtle intergroup variations.

### Limitations and Future Directions

This study has some limitations. First, it employed the reverse correlation method to identify potential differences between groups. No corresponding statistical values were generated because this approach does not rely on hypothesis testing and instead uses reverse inference from predictive models to uncover possible points of divergence. The accuracy of the differences identified is therefore dependent on the generalisation capability of the model. Second, owing to the small sample size, overfitting was inevitable during neural network training, which reduced the generalisability of the final model. As a result, the findings may not be readily extrapolated to broader populations. We are conducting further in-depth research on this method and cross-validating it with other statistical approaches. In subsequent studies, we aim to establish a statistically meaningful minimum sample size for this method.

Nevertheless, the research framework proposed in this study effectively addresses the challenges in detecting subtle intergroup differences when sample sizes are limited. Based on its methodological principles, we hypothesise that as sample sizes increase and the predictive performance of neural network training improves, the conclusions derived may generalise more effectively to real-world scenarios than those obtained using traditional statistical methods, thereby underscoring the value of this approach and its potential for further investigation. Further, the framework is not confined to multilayer neural networks. Given the emergence of models that provide better machine learning-based training performance at lower computational costs, the reverse correlation method proposed here could serve as a simpler alternative for research exploration.

## 5. Conclusion

This study utilised multilayer neural networks combined with a reverse correlation approach, demonstrating that LOD-MCI may be linked to structural alterations in the temporal lobe, thereby providing valuable insights into its pathological mechanisms. Importantly, the proposed framework illuminates the decision-making logic of multilayer neural networks and effectively tackles the difficulty of detecting subtle inter-group differences when sample sizes are limited. Further, the reverse correlation method is not specific to neural networks and can be applied across other domains. Given the growing accessibility of high-performance and cost-efficient machine learning models, the reverse correlation technique introduced in this study presents a practical and accessible alternative for exploratory research in the field.

## Availability of Data and Materials

The data and materials used in this study are available from the corresponding author upon reasonable request.

## Supplementary Material

Supplementary material associated with this article can be found, in the online version, at https://doi.org/10.31083/AP44585.
