# Supplementary material for: Structural Alterations and Cognitive Impairment in Late-Onset Depression: A Reverse Correlation Analysis
Source: Alpha Psychiatry. 2026 Feb 3;27(1):44585. doi: 10.31083/AP44585 (PMC12957978; doi:10.31083/AP44585)
Supplement: Supplementary file 1 [file 2757-8038-27-1-44585-s1.zip › Supplementary Material.docx]

**Supplementary Table 1 The medication history of LOD**

|  | LOD |
| --- | --- |
|  | N=41 |
| Antidepressants |  |
| No antidepressants | 17 |
| SNRI | 9 |
| SSRI | 11 |
| TCAs | 0 |
| NaSSA | 4 |
| Benzodiazepines |  |
| With | 21 |
| Without | 20 |


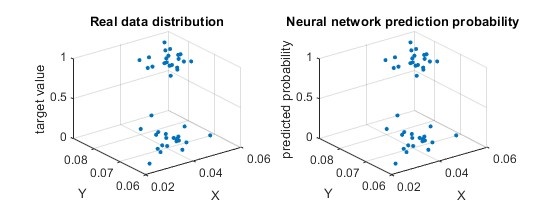


**Supplementary Figure 1:** 3D distribution of real data and predicted probabilities

The "Real data distribution" on the left is the 3D scatter plot of real data on features (\(X, Y\)) and target values (\(target\ value\)); the "Neural network prediction probability" on the right is the predicted probability distribution of each sample by the model. The similarity in distribution patterns between the two indicates that the model has learned the feature correlations of the data.


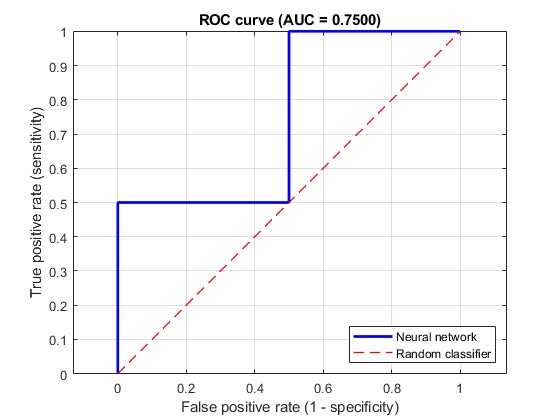


**Supplementary Figure 2: ROC curve:**

ROC curve: The horizontal axis represents the False Positive Rate (FPR, (1 - specificity)) and the vertical axis represents the True Positive Rate (TPR, sensitivity). The closer the curve is to the upper left corner, the stronger the model's classification ability. AUC (Area Under the Curve)=0.75, which is higher than that of the "Random Classifier" (red dashed line, AUC=0.5). This indicates that the model can effectively distinguish between positive and negative samples, but there is still room for improvement towards "perfect classification" (AUC=1). (The current study has a limited sample size, and this ROC curve represents the result of a single sampling trial. In subsequent research with an increased sample size, we will augment the number of repeated sampling trials and obtain confidence intervals for the ROC curves.)

**Supplementary Material 1**

Schematic diagram of the difference node matrix for LOD-MCI in structural networks: After n=10,000 cyclic iterations, 2,645 statistically significant difference matrices were identified. These matrices were averaged to produce the following schematic. The color represents the frequency, ranging from white to dark blue, where a darker shade indicates a higher occurrence rate of the connection in the selected random matrices.
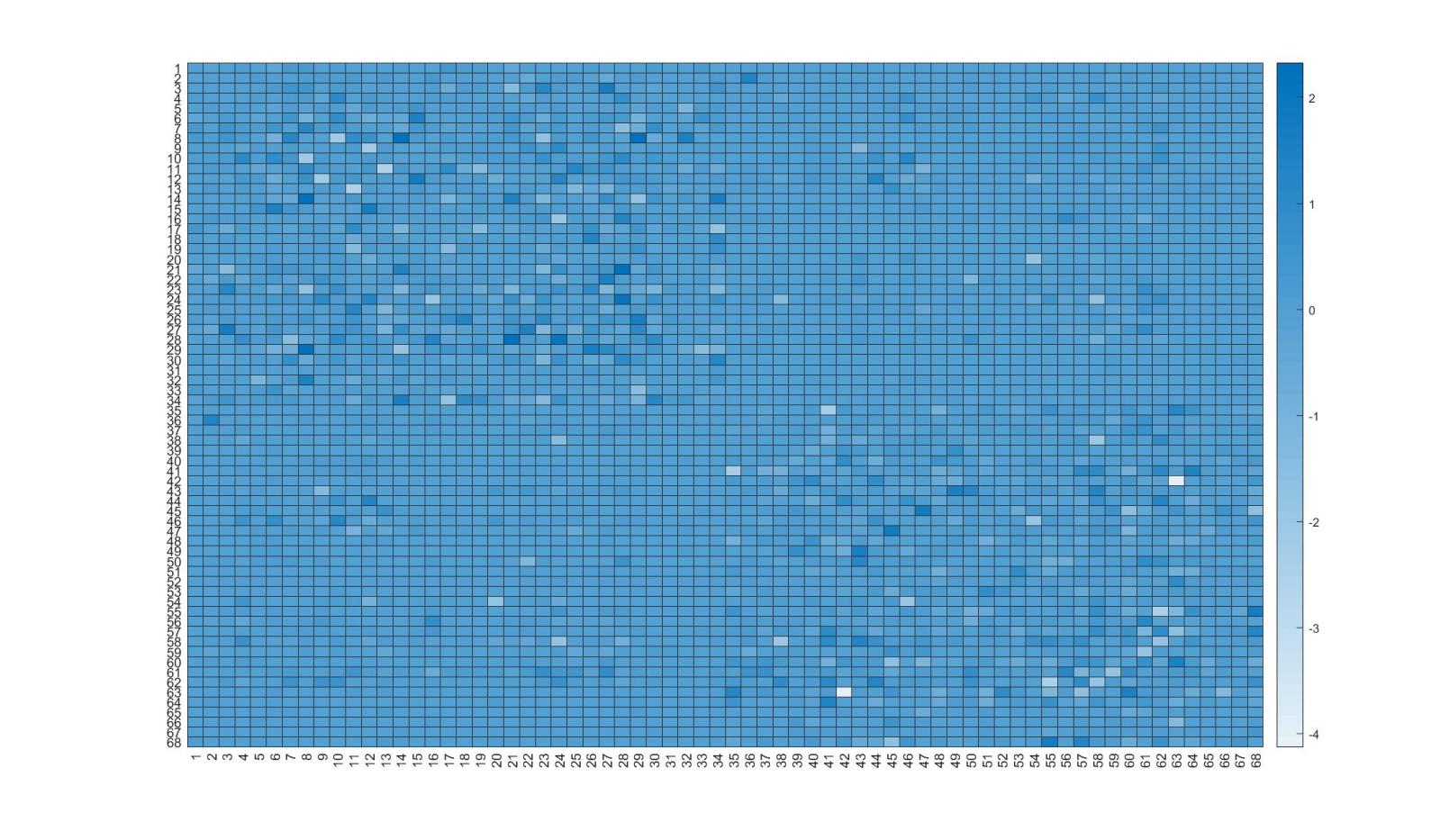


where the top 5% strongest connections are represented as: lh.inferiortemporal-lh.superiortemporal, lh.inferiortemporal-lh.middletemporal, lh.postcentral-lh.superiorparietal, lh.precuneus-lh.superiorparietal, rh.lateralorbitofrontal-rh.medialorbitofrontal, rh.postcentral-rh.insula, lh.caudalmiddlefrontal-lh.superiorfrontal, lh.lingual-lh.parahippocampal, rh.isthmuscingulate-rh.parahippocampal, lh.rostralmiddlefrontal-lh.superiortemporal, lh.middletemporal-lh.insula, lh.fusiform-lh.parahippocampal, rh.rostralmiddlefrontal-rh.superiortemporal, lh.inferiortemporal-lh.temporalpole, lh.posteriorcingulate-lh.superiorfrontal, rh.inferiorparietal-rh.supramarginal, lh.caudalanteriorcingulate-rh.caudalanteriorcingulate, lh.middletemporal-lh.postcentral, lh.lingual-rh.lateraloccipital , rh.precentral-rh.insula, lh.parsorbitalis-lh.rostralmiddlefrontal, rh.isthmuscingulate-rh.precuneus, lh.paracentral-lh.superiorparietal, rh.lateraloccipital -rh.superiorparietal, lh.inferiorparietal-lh.inferiortemporal, lh.lateralorbitofrontal-lh.rostralanteriorcingulate, lh.lingual-lh.precuneus, rh.posteriorcingulate-rh.superiorfrontal, lh.caudalmiddlefrontal-lh.precentral, lh.lateraloccipital -rh.lingual, lh.supramarginal-lh.insula, rh.isthmuscingulate-rh.paracentral, rh.bankssts-rh.superiortemporal, lh.cuneus-lh.lateraloccipital , lh.superiorfrontal-lh.superiortemporal, rh.inferiorparietal-rh.superiorparietal, lh.lateraloccipital -lh.superiorparietal, rh.inferiorparietal-rh.precuneus, lh.parsorbitalis-lh.insula, lh.superiorparietal-lh.supramarginal, rh.cuneus-rh.superiorparietal, lh.isthmuscingulate-lh.precuneus, rh.parsorbitalis-rh.superiortemporal, rh.inferiorparietal-rh.precentral, lh.precentral-lh.rostralmiddlefrontal, rh.fusiform-rh.inferiortemporal, rh.inferiortemporal-rh.lateraloccipital , rh.parsopercularis-rh.parstriangularis, lh.paracentral-rh.posteriorcingulate, lh.fusiform-lh.lateraloccipital , lh.superiorfrontal-rh.superiorfrontal, lh.lateralorbitofrontal-lh.parsopercularis, rh.precentral-rh.superiorparietal, rh.paracentral-rh.superiorfrontal, lh.inferiortemporal-lh.lateralorbitofrontal, rh.rostralmiddlefrontal-rh.superiorfrontal, rh.postcentral-rh.precuneus, lh.fusiform-rh.lingual, rh.precentral-rh.precuneus, lh.middletemporal-lh.superiorfrontal, lh.cuneus-lh.superiorparietal, lh.precentral-rh.superiorfrontal, rh.entorhinal-rh.parahippocampal, lh.inferiorparietal-lh.supramarginal, rh.paracentral-rh.superiorparietal, rh.lateraloccipital -rh.precuneus, rh.postcentral-rh.supramarginal, lh.parstriangularis-lh.insula, lh.medialorbitofrontal-rh.lateralorbitofrontal, lh.postcentral-lh.precuneus, rh.superiorparietal-rh.insula, rh.caudalmiddlefrontal-rh.superiorfrontal, lh.precuneus-rh.superiorparietal, rh.lateraloccipital -rh.lingual, lh.parsopercularis-lh.rostralmiddlefrontal, lh.lateraloccipital -lh.precentral, lh.inferiortemporal-lh.lingual, lh.cuneus-rh.precuneus, lh.precuneus-lh.supramarginal, lh.fusiform-lh.transversetemporal, rh.pericalcarine-rh.precuneus, lh.isthmuscingulate-rh.superiorparietal, rh.bankssts-rh.postcentral, rh.lateralorbitofrontal-rh.rostralanteriorcingulate, lh.lateraloccipital -rh.lateraloccipital , lh.precuneus-lh.insula, lh.superiorparietal-rh.paracentral, rh.fusiform-rh.middletemporal, lh.parstriangularis-lh.superiortemporal, rh.precentral-rh.rostralmiddlefrontal, rh.bankssts-rh.supramarginal, rh.lateralorbitofrontal-rh.temporalpole, rh.caudalanteriorcingulate-rh.superiorfrontal, lh.cuneus-rh.lingual, lh.isthmuscingulate-lh.posteriorcingulate, lh.lateraloccipital -rh.superiorparietal, lh.inferiorparietal-rh.superiorparietal, lh.precentral-lh.precuneus, lh.parsopercularis-lh.superiortemporal, rh.caudalmiddlefrontal-rh.rostralmiddlefrontal, lh.caudalmiddlefrontal-lh.inferiorparietal, lh.entorhinal-lh.parahippocampal, lh.lateralorbitofrontal-lh.superiorfrontal, rh.precuneus-rh.rostralmiddlefrontal, lh.cuneus-rh.pericalcarine, lh.rostralmiddlefrontal-lh.superiorfrontal, rh.entorhinal-rh.inferiortemporal, lh.caudalmiddlefrontal-lh.inferiortemporal, lh.caudalmiddlefrontal-lh.insula, lh.precuneus-rh.postcentral, lh.lateralorbitofrontal-lh.rostralmiddlefrontal, lh.middletemporal-lh.supramarginal, lh.fusiform-lh.inferiorparietal, rh.lingual-rh.insula, lh.postcentral-lh.superiorfrontal, lh.postcentral-lh.rostralmiddlefrontal, lh.fusiform-lh.postcentral, rh.precuneus-rh.supramarginal, rh.lateralorbitofrontal-rh.superiortemporal, lh.lateralorbitofrontal-rh.superiorfrontal, rh.bankssts-rh.rostralmiddlefrontal, lh.rostralanteriorcingulate-rh.superiorfrontal, rh.inferiortemporal-rh.insula, rh.medialorbitofrontal-rh.rostralanteriorcingulate, lh.lateraloccipital -lh.superiorfrontal, lh.inferiorparietal-lh.postcentral, lh.superiorparietal-lh.superiortemporal, lh.fusiform-lh.superiorfrontal, rh.inferiortemporal-rh.rostralmiddlefrontal, lh.lateralorbitofrontal-lh.lingual, lh.pericalcarine-rh.isthmuscingulate, rh.transversetemporal-rh.insula, rh.posteriorcingulate-rh.precuneus, lh.postcentral-lh.supramarginal, lh.pericalcarine-rh.lingual, lh.superiortemporal-lh.supramarginal, lh.lateraloccipital -lh.supramarginal, rh.superiorfrontal-rh.supramarginal, rh.entorhinal-rh.lateralorbitofrontal, rh.parsorbitalis-rh.parstriangularis, rh.medialorbitofrontal-rh.precuneus, lh.paracentral-lh.precentral, lh.inferiorparietal-lh.parahippocampal, lh.inferiorparietal-lh.lateraloccipital , lh.inferiortemporal-lh.rostralmiddlefrontal, lh.posteriorcingulate-rh.isthmuscingulate, lh.precuneus-rh.superiorfrontal, lh.bankssts-lh.parsopercularis, lh.paracentral-rh.precentral, lh.lingual-lh.superiorparietal, lh.temporalpole-lh.insula, rh.lateraloccipital -rh.parsopercularis, rh.bankssts-rh.superiorparietal, rh.isthmuscingulate-rh.pericalcarine, lh.medialorbitofrontal-lh.parahippocampal, lh.bankssts-lh.inferiorparietal, rh.bankssts-rh.parsopercularis, lh.fusiform-lh.superiorparietal, rh.entorhinal-rh.temporalpole, rh.caudalanteriorcingulate-rh.rostralmiddlefrontal, lh.isthmuscingulate-rh.posteriorcingulate, lh.rostralanteriorcingulate-lh.rostralmiddlefrontal, rh.inferiorparietal-rh.postcentral, lh.lingual-lh.superiortemporal, lh.caudalanteriorcingulate-lh.paracentral, rh.precuneus-rh.insula, rh.fusiform-rh.pericalcarine, lh.cuneus-lh.lingual, lh.superiorfrontal-rh.rostralmiddlefrontal, lh.inferiortemporal-lh.postcentral, rh.fusiform-rh.insula, lh.fusiform-lh.precuneus, rh.bankssts-rh.inferiortemporal, lh.inferiorparietal-lh.precuneus, lh.rostralmiddlefrontal-lh.frontalpole, rh.parstriangularis-rh.postcentral, lh.lateralorbitofrontal-lh.middletemporal, lh.lingual-rh.fusiform, rh.fusiform-rh.parahippocampal, rh.cuneus-rh.precentral, rh.inferiorparietal-rh.isthmuscingulate, lh.bankssts-lh.inferiortemporal, lh.inferiorparietal-lh.insula, rh.inferiorparietal-rh.superiorfrontal, lh.rostralmiddlefrontal-lh.superiorparietal, lh.superiorparietal-rh.isthmuscingulate, lh.parahippocampal-lh.superiorparietal, lh.bankssts-lh.transversetemporal, rh.lateralorbitofrontal-rh.pericalcarine, rh.parahippocampal-rh.precuneus, lh.paracentral-lh.superiortemporal, lh.lateraloccipital -lh.postcentral, rh.cuneus-rh.rostralmiddlefrontal, rh.isthmuscingulate-rh.postcentral, lh.middletemporal-lh.temporalpole, rh.lingual-rh.postcentral, rh.caudalmiddlefrontal-rh.superiortemporal, lh.posteriorcingulate-rh.superiorfrontal, rh.caudalmiddlefrontal-rh.insula, rh.entorhinal-rh.isthmuscingulate, rh.parstriangularis-rh.supramarginal, lh.paracentral-rh.postcentral, rh.cuneus-rh.medialorbitofrontal, lh.caudalmiddlefrontal-lh.lateraloccipital , rh.superiorparietal-rh.superiortemporal, lh.lateraloccipital -lh.lingual, rh.middletemporal-rh.parstriangularis, lh.caudalanteriorcingulate-lh.medialorbitofrontal, lh.paracentral-lh.insula, rh.lateraloccipital -rh.parahippocampal, rh.superiorfrontal-rh.superiortemporal, lh.caudalanteriorcingulate-lh.lateralorbitofrontal, lh.lateraloccipital -rh.precuneus, rh.parsopercularis-rh.rostralmiddlefrontal, lh.middletemporal-lh.parstriangularis, lh.entorhinal-rh.lateralorbitofrontal, lh.inferiorparietal-lh.isthmuscingulate, lh.lingual-lh.insula, lh.inferiorparietal-rh.paracentral, lh.fusiform-lh.posteriorcingulate, lh.medialorbitofrontal-rh.frontalpole, lh.superiorfrontal-rh.caudalmiddlefrontal, rh.caudalanteriorcingulate-rh.isthmuscingulate, lh.caudalmiddlefrontal-rh.superiorparietal, lh.bankssts-lh.supramarginal, lh.lateraloccipital -rh.cuneus, rh.lateraloccipital -rh.rostralmiddlefrontal, lh.caudalmiddlefrontal-lh.rostralmiddlefrontal, lh.parahippocampal-lh.pericalcarine, rh.middletemporal-rh.insula, rh.parsorbitalis-rh.precentral, rh.caudalanteriorcingulate-rh.precuneus

Schematic diagram of the difference node matrix for LOD-NON-MCI in structural networks: After n=10,000 cyclic iterations, 118 statistically significant difference matrices were identified. These matrices were averaged to produce the following schematic. The color represents the frequency, ranging from white to dark blue, where a darker shade indicates a higher occurrence rate of the connection in the selected random matrices.
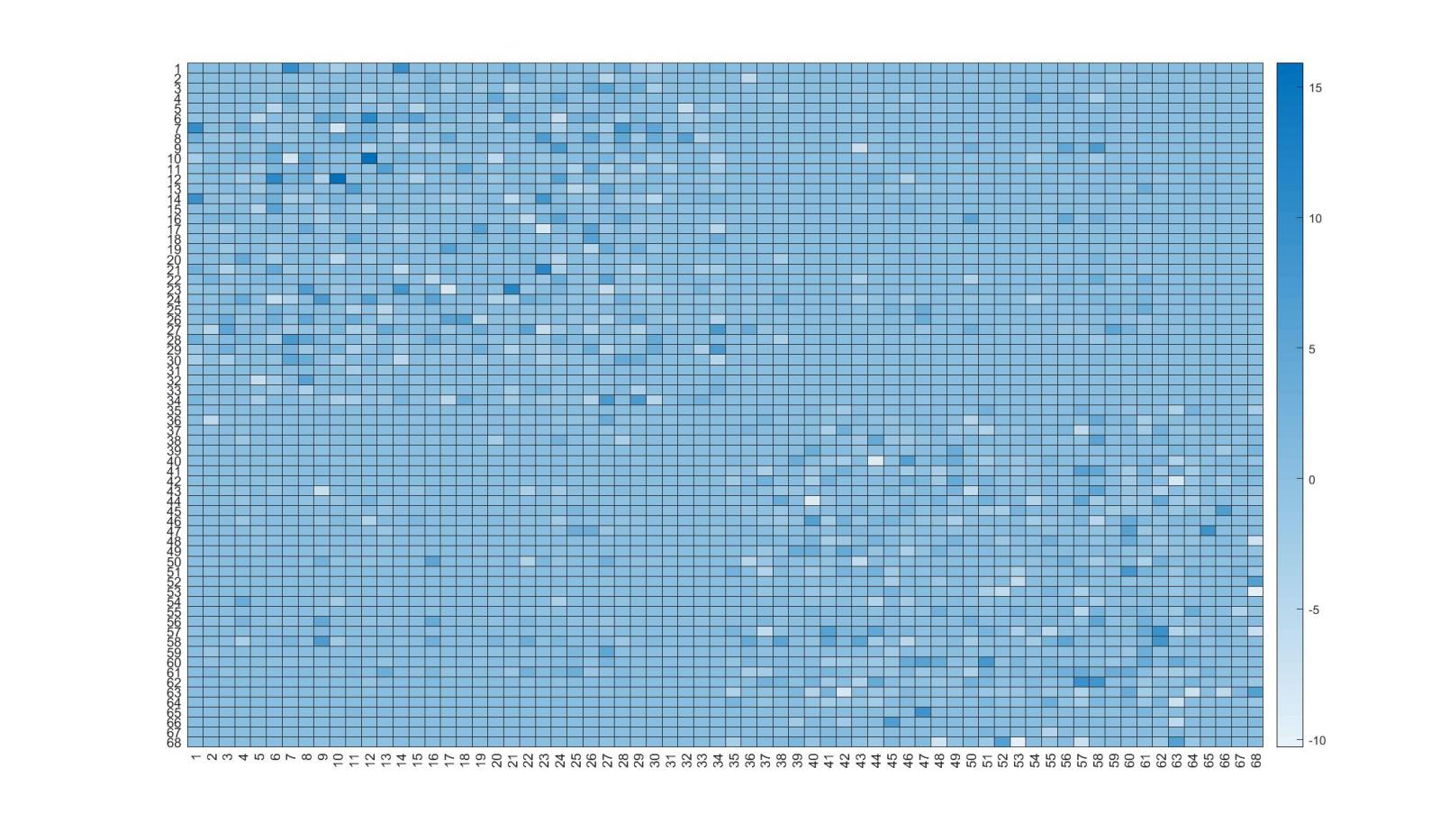


where the top 5% strongest connections are represented as: lh.lingual-lh.lateraloccipital , lh.precentral-lh.postcentral, lh.lingual-lh.fusiform, lh.inferiorparietal-lh.bankssts, rh.superiorparietal-rh.precentral, lh.middletemporal-lh.bankssts, rh.frontalpole-rh.medialorbitofrontal, rh.superiorparietal-rh.precuneus, lh.precentral-lh.middletemporal, lh.superiorparietal-lh.inferiorparietal, rh.rostralmiddlefrontal-rh.parsopercularis, lh.insula-lh.superiorfrontal, rh.precuneus-lh.isthmuscingulate, lh.insula-lh.superiortemporal, lh.precentral-lh.inferiortemporal, lh.medialorbitofrontal-lh.lateralorbitofrontal, lh.precuneus-lh.isthmuscingulate, rh.temporalpole-rh.lateralorbitofrontal, rh.insula-rh.superiortemporal, rh.insula-rh.parsorbitalis, rh.rostralmiddlefrontal-rh.medialorbitofrontal, rh.lingual-rh.fusiform, lh.parstriangularis-lh.parsopercularis, lh.precuneus-lh.paracentral, lh.precuneus-lh.lingual, rh.precentral-rh.inferiorparietal, rh.precuneus-rh.cuneus, lh.rostralmiddlefrontal-lh.parsorbitalis, rh.paracentral-lh.paracentral, lh.temporalpole-lh.inferiortemporal, lh.rostralmiddlefrontal-lh.parsopercularis, lh.parahippocampal-lh.fusiform, rh.precuneus-rh.posteriorcingulate, lh.superiorfrontal-lh.posteriorcingulate, rh.rostralanteriorcingulate-lh.superiorfrontal, lh.supramarginal-lh.inferiorparietal, rh.posteriorcingulate-lh.isthmuscingulate, rh.precuneus-rh.isthmuscingulate, rh.precuneus-rh.inferiorparietal, lh.superiorparietal-lh.inferiortemporal, lh.superiorfrontal-lh.caudalmiddlefrontal, rh.parahippocampal-rh.inferiortemporal, rh.precentral-rh.lateraloccipital , lh.rostralmiddlefrontal-lh.inferiortemporal, rh.lateraloccipital -rh.cuneus, lh.parsorbitalis-lh.lateralorbitofrontal, lh.pericalcarine-lh.cuneus, lh.isthmuscingulate-lh.fusiform, lh.supramarginal-lh.inferiortemporal, rh.precuneus-rh.caudalanteriorcingulate, lh.postcentral-lh.fusiform, rh.posteriorcingulate-lh.paracentral, lh.lateraloccipital -lh.inferiortemporal, lh.supramarginal-lh.superiorparietal, rh.caudalanteriorcingulate-lh.superiorfrontal, rh.superiorfrontal-rh.rostralanteriorcingulate, lh.parsopercularis-lh.inferiortemporal, lh.superiorfrontal-lh.medialorbitofrontal, rh.superiorparietal-rh.lateraloccipital , rh.parahippocampal-rh.entorhinal, rh.rostralmiddlefrontal-rh.middletemporal, rh.superiorfrontal-rh.precentral, rh.superiorfrontal-rh.rostralmiddlefrontal, rh.rostralmiddlefrontal-rh.lingual, rh.pericalcarine-lh.cuneus, lh.precuneus-lh.posteriorcingulate, lh.precuneus-lh.cuneus, rh.precuneus-lh.posteriorcingulate, rh.superiortemporal-rh.rostralmiddlefrontal, rh.parahippocampal-rh.fusiform, rh.superiorfrontal-lh.rostralanteriorcingulate, lh.superiorfrontal-lh.parstriangularis, lh.rostralmiddlefrontal-lh.lateralorbitofrontal, rh.supramarginal-rh.postcentral, rh.medialorbitofrontal-lh.rostralanteriorcingulate, rh.fusiform-rh.entorhinal, rh.medialorbitofrontal-lh.rostralmiddlefrontal, lh.lateraloccipital -lh.fusiform, rh.superiorfrontal-lh.medialorbitofrontal, lh.superiorparietal-lh.paracentral, lh.supramarginal-lh.superiortemporal, lh.rostralmiddlefrontal-lh.caudalmiddlefrontal, rh.postcentral-rh.middletemporal, lh.inferiortemporal-lh.bankssts, lh.postcentral-lh.bankssts, rh.inferiortemporal-rh.caudalmiddlefrontal, lh.insula-lh.parsorbitalis, lh.rostralmiddlefrontal-lh.fusiform, rh.paracentral-lh.superiorfrontal, lh.superiortemporal-lh.rostralmiddlefrontal, lh.middletemporal-lh.fusiform, lh.lingual-lh.inferiortemporal, lh.superiorparietal-lh.bankssts, lh.lateralorbitofrontal-lh.inferiortemporal, rh.precuneus-lh.paracentral, lh.superiortemporal-lh.caudalmiddlefrontal, rh.superiorfrontal-rh.posteriorcingulate, rh.inferiortemporal-rh.inferiorparietal, rh.lingual-rh.inferiortemporal, lh.superiortemporal-lh.parstriangularis, lh.insula-lh.transversetemporal, rh.cuneus-lh.precuneus, rh.posteriorcingulate-rh.paracentral, rh.parsopercularis-rh.bankssts, rh.parsopercularis-rh.lateraloccipital , lh.inferiorparietal-lh.cuneus, rh.superiorfrontal-lh.posteriorcingulate, rh.paracentral-lh.isthmuscingulate, rh.superiorparietal-rh.caudalmiddlefrontal, rh.parstriangularis-rh.lateralorbitofrontal, lh.superiorparietal-lh.precuneus, rh.middletemporal-rh.lateraloccipital , rh.precentral-rh.parsopercularis, lh.posteriorcingulate-lh.caudalanteriorcingulate, rh.superiorparietal-rh.paracentral, rh.parsopercularis-rh.inferiortemporal, lh.superiortemporal-lh.inferiorparietal, rh.rostralmiddlefrontal-lh.superiorfrontal, rh.supramarginal-rh.bankssts, lh.medialorbitofrontal-lh.fusiform, rh.posteriorcingulate-lh.cuneus, rh.precuneus-rh.postcentral, rh.superiortemporal-rh.inferiorparietal, lh.parstriangularis-lh.parsorbitalis, lh.superiorparietal-lh.isthmuscingulate, rh.precentral-rh.bankssts, lh.rostralanteriorcingulate-lh.fusiform, rh.superiorparietal-rh.cuneus, rh.superiorfrontal-rh.parsopercularis, rh.superiorparietal-rh.fusiform, rh.parahippocampal-rh.middletemporal, rh.lateralorbitofrontal-lh.rostralanteriorcingulate, lh.transversetemporal-lh.precentral, lh.precentral-lh.paracentral, rh.supramarginal-rh.superiorparietal, lh.superiorfrontal-lh.lateraloccipital , lh.superiortemporal-lh.lateraloccipital , rh.lateraloccipital -lh.lingual, lh.postcentral-lh.parsopercularis, rh.superiorfrontal-rh.inferiorparietal, lh.paracentral-lh.caudalanteriorcingulate, rh.caudalmiddlefrontal-rh.caudalanteriorcingulate, rh.precuneus-lh.pericalcarine, rh.parahippocampal-rh.isthmuscingulate, rh.posteriorcingulate-rh.postcentral, rh.superiorfrontal-lh.precuneus, rh.rostralanteriorcingulate-lh.rostralanteriorcingulate, lh.superiortemporal-lh.fusiform, lh.insula-lh.bankssts, rh.precuneus-lh.precuneus, rh.postcentral-rh.parstriangularis, rh.frontalpole-rh.superiorfrontal, rh.precuneus-lh.inferiorparietal, lh.lingual-lh.inferiorparietal, rh.insula-rh.entorhinal, lh.middletemporal-lh.lateraloccipital , rh.lingual-rh.lateralorbitofrontal, rh.transversetemporal-rh.parsopercularis, rh.rostralmiddlefrontal-rh.parstriangularis, rh.middletemporal-rh.fusiform, lh.lateraloccipital -lh.cuneus, rh.supramarginal-rh.parsopercularis, lh.precuneus-lh.precentral, rh.superiorparietal-rh.parsopercularis, rh.supramarginal-rh.rostralmiddlefrontal, lh.precuneus-lh.parahippocampal, rh.precentral-rh.lingual, lh.superiorparietal-lh.cuneus, rh.lateralorbitofrontal-rh.isthmuscingulate, rh.superiorfrontal-lh.paracentral, rh.rostralmiddlefrontal-rh.postcentral, lh.paracentral-lh.caudalmiddlefrontal, lh.superiortemporal-lh.medialorbitofrontal, lh.parsopercularis-lh.fusiform, rh.insula-rh.lateralorbitofrontal, rh.medialorbitofrontal-rh.inferiortemporal, rh.precentral-rh.inferiortemporal, rh.posteriorcingulate-rh.caudalmiddlefrontal, lh.insula-lh.middletemporal, rh.superiortemporal-rh.entorhinal, lh.superiorfrontal-lh.middletemporal, rh.lingual-lh.parahippocampal, rh.postcentral-rh.fusiform, rh.postcentral-rh.paracentral, lh.postcentral-lh.parstriangularis, lh.lateralorbitofrontal-lh.fusiform, rh.insula-rh.inferiorparietal, rh.paracentral-lh.precentral, rh.precentral-rh.isthmuscingulate, rh.posteriorcingulate-lh.posteriorcingulate, rh.superiorfrontal-rh.postcentral, rh.superiorfrontal-rh.precuneus, lh.posteriorcingulate-lh.pericalcarine, lh.temporalpole-lh.superiortemporal, rh.rostralmiddlefrontal-rh.caudalanteriorcingulate, lh.parahippocampal-lh.medialorbitofrontal, rh.insula-rh.medialorbitofrontal, lh.parstriangularis-lh.inferiortemporal, rh.superiorfrontal-lh.caudalanteriorcingulate, rh.rostralmiddlefrontal-lh.rostralanteriorcingulate, rh.supramarginal-rh.precuneus, rh.transversetemporal-rh.inferiorparietal, rh.lingual-rh.lateraloccipital , lh.insula-lh.temporalpole, lh.rostralmiddlefrontal-lh.caudalanteriorcingulate, lh.transversetemporal-lh.superiorparietal, lh.precentral-lh.isthmuscingulate, lh.frontalpole-lh.superiorfrontal, rh.parsopercularis-rh.middletemporal, rh.medialorbitofrontal-rh.isthmuscingulate, rh.middletemporal-rh.caudalmiddlefrontal, rh.pericalcarine-rh.cuneus, lh.superiorfrontal-lh.paracentral, rh.superiortemporal-rh.lingual, rh.superiorfrontal-rh.parstriangularis, rh.insula-rh.temporalpole, rh.rostralanteriorcingulate-rh.caudalanteriorcingulate, lh.posteriorcingulate-lh.entorhinal, rh.parstriangularis-rh.lateraloccipital , lh.precentral-lh.parstriangularis, rh.precuneus-rh.precentral, rh.precuneus-lh.supramarginal, rh.precentral-rh.middletemporal, lh.parahippocampal-lh.inferiortemporal, rh.postcentral-rh.inferiortemporal, lh.superiorfrontal-lh.entorhinal, rh.medialorbitofrontal-lh.lateralorbitofrontal, rh.insula-rh.cuneus, rh.postcentral-rh.isthmuscingulate, lh.posteriorcingulate-lh.medialorbitofrontal, rh.superiorparietal-rh.pericalcarine, lh.paracentral-lh.lateraloccipital

**Supplementary Material 2**

Node region information

| Lable | Region |
| --- | --- |
| 1 | l.bankssts |
| 2 | l.caudalanteriorcingulate |
| 3 | l.caudalmiddlefrontal |
| 4 | l.cuneus |
| 5 | l.entorhinal |
| 6 | l.fusiform |
| 7 | l.inferiorparietal |
| 8 | l.inferiortemporal |
| 9 | l.isthmuscingulate |
| 10 | l.lateraloccipital |
| 11 | l.lateralorbitofrontal |
| 12 | l.lingual |
| 13 | l.medialorbitofrontal |
| 14 | l.middletemporal |
| 15 | l.parahippocampal |
| 16 | l.paracentral |
| 17 | l.parsopercularis |
| 18 | l.parsorbitalis |
| 19 | l.parstriangularis |
| 20 | l.pericalcarine |
| 21 | l.postcentral |
| 22 | l.posteriorcingulate |
| 23 | l.precentral |
| 24 | l.precuneus |
| 25 | l.rostralanteriorcingulate |
| 26 | l.rostralmiddlefrontal |
| 27 | l.superiorfrontal |
| 28 | l.superiorparietal |
| 29 | l.superiortemporal |
| 30 | l.supramarginal |
| 31 | l.frontalpole |
| 32 | l.temporalpole |
| 33 | l.transversetemporal |
| 34 | l.insula |
| 35 | r.bankssts |
| 36 | r.caudalanteriorcingulate |
| 37 | r.caudalmiddlefrontal |
| 38 | r.cuneus |
| 39 | r.entorhinal |
| 40 | r.fusiform |
| 41 | r.inferiorparietal |
| 42 | r.inferiortemporal |
| 43 | r.isthmuscingulate |
| 44 | r.lateraloccipital |
| 45 | r.lateralorbitofrontal |
| 46 | r.lingual |
| 47 | r.medialorbitofrontal |
| 48 | r.middletemporal |
| 49 | r.parahippocampal |
| 50 | r.paracentral |
| 51 | r.parsopercularis |
| 52 | r.parsorbitalis |
| 53 | r.parstriangularis |
| 54 | r.pericalcarine |
| 55 | r.postcentral |
| 56 | r.posteriorcingulate |
| 57 | r.precentral |
| 58 | r.precuneus |
| 59 | r.rostralanteriorcingulate |
| 60 | r.rostralmiddlefrontal |
| 61 | r.superiorfrontal |
| 62 | r.superiorparietal |
| 63 | r.superiortemporal |
| 64 | r.supramarginal |
| 65 | r.frontalpole |
| 66 | r.temporalpole |
| 67 | r.transversetemporal |
| 68 | r.insula |
